# Systemic metastases in large cell neuroendocrine prostate cancer: a rare case report and literature review

**DOI:** 10.3389/fonc.2024.1398673

**Published:** 2024-05-15

**Authors:** Maolin Xiao, Wei Tong, Xiao Xiao, Xiaofeng Pu, Faxian Yi

**Affiliations:** Department of Urology, Chongqing General Hospital, Chongqing University, Chongqing, China

**Keywords:** prostate cancer, large cell, neuroendocrine carcinoma, metastasis, transdifferentiation

## Abstract

Neuroendocrine prostate neoplasms, encompassing small cell carcinoma, carcinoid, and large cell carcinoma, are infrequently observed in malignant prostate tumors. The occurrence of large cell neuroendocrine prostate cancer (LCNEPC) is exceedingly rare. In this study, the patient initially presented with a persistent dysuria for a duration of one year, accompanied by a serum prostate-specific antigen (PSA) level of 17.83ng/mL. Prostate magnetic resonance imaging (MRI) and chest computed tomography (CT) scan showed that a neoplastic lesion was considered, and prostate biopsy confirmed prostate adenocarcinoma with a Gleason score of 7 (4 + 3). Then, thoracoscopic lung tumor resection was performed, and the pathological examination revealed the presence of primary moderately differentiated invasive adenocarcinoma of the lung and metastatic prostate adenocarcinoma, the Gleason score was 8 (4 + 4). After 1 year of endocrine therapy with goserelin acetate and bicalutamide, he underwent a laparoscopic radical prostatectomy (LRP), the pathological report indicated the presence of adenocarcinoma mixed with NE carcinoma. Two months after the LRP, the patient experienced gross hematuria and sacral tail pain. Further examination revealed multiple metastatic lesions throughout the body. He also underwent transurethral resection of bladder tumor (TURBT) for bladder tumor and received etoposide+ cisplatin chemotherapy three weeks post-surgery. The patient eventually died of multi-organ failure due to myelosuppression after chemotherapy. This case report presents an uncommon instance of LCNEPC with widespread systemic metastases, while also providing a comprehensive review of existing literature to facilitate improved management and treatment strategies for similar patients in subsequent cases.

## 1 Introduction

Prostate cancer (Pca) exhibits the highest incidence rate among males worldwide annually and ranks as the second most prevalent cause of tumor-associated mortality (1). Neuroendocrine prostate cancer (NEPC) is an uncommon subtype of prostate malignancy, primarily originating from prostate adenocarcinoma (PRAD), and progresses to mixed neuroendocrine (NE) carcinoma-acinar adenocarcinomas (2). In 2016, the World Health Organization (WHO) categorized prostate neuroendocrine tumors as highly differentiated carcinoid tumors, small cell neuroendocrine carcinoma (SCNC), and large cell neuroendocrine prostate cancer (LCNEPC) (3). Among these subtypes, carcinoid tumors and LCNEPC are less prevalent than SCNC (3, 4). The majority of reported cases of LCNEPC have been observed in patients with adenocarcinoma who have undergone long-term androgen deprivation therapy (ADT), indicating transdifferentiation. However, primary LCNEPC cases have also been reported in several studies (5, 6), although they are less common than cases with adenocarcinoma transdifferentiation. LCNEPC exhibits high invasiveness and is frequently associated with extensive metastasis, resulting in a poor prognosis (7, 8). The primary treatment for LCNEPC is chemotherapy, with the main regimen being etoposide+ platinum (9). Previous reports have indicated that the combination of etoposide with either cisplatin or carboplatin is generally effective (10, 11). In this case, the patient initially presented with progressive dysuria, and PRAD and lung tumors were diagnosed during hospitalization. While receiving goserelin acetate and bicalutamide endocrine therapy, the patient underwent thoracoscopic lung tumor resection, and postoperative pathological diagnosis suggested primary lung adenocarcinoma along with metastases of PRAD. He subsequently received 4 cycles of sintilimab, paclitaxel, and loplatin immunotherapy combined with chemotherapy. After 1 year of ADT treatment he underwent laparoscopic radical prostatectomy (LRP) for prostate cancer and was diagnosed with LCNEPC at this time. 2 months after the operation, the patient developed bladder, lung, liver, pelvic, and multiple bone metastases and recurrence, and received etoposide and cisplatin chemotherapy. After 1 cycle of treatment, the patient died due to multi-organ dysfunction caused by severe myelosuppression.

## 2 Case description

**Figure 1:**
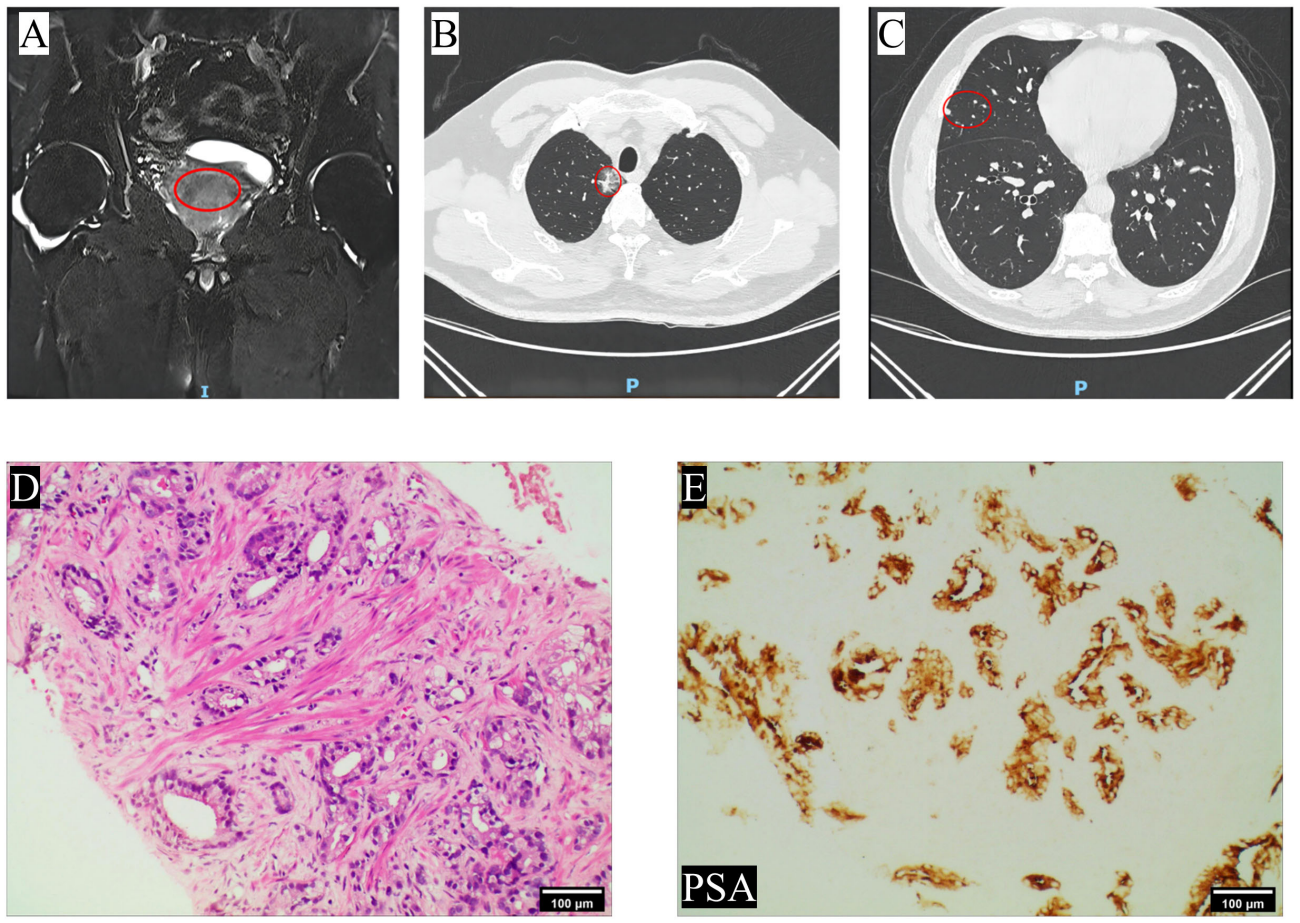
Imaging and pathology findings at the patient’s first visit. **(A)** The prostate MRI at T2-weighted images revealed the presence of nodular patchy hypointense lesion in the migratory zone in the direction of points 9–11; **(B)** Chest CT showed a solid nodule was observed in the apical segment of the upper lobe of the right lung, which was considered a primary lesion; **(C)** scattered solid nodules were observed in the middle lobe of the right lung, suggesting metastasis; **(D)** HE staining. The glands exhibit irregular and fused, or small infiltrating glands with varying shapes and prominent nucleoli (4×); **(E)** Immunohistochemical staining revealed positive expression of PSA (10×). The site of the lesion was marked with a red circle.

The 65-year-old patient was admitted to the department of urology on March 14th, 2022 due to progressive dysuria. The patient did not exhibit symptoms such as gross hematuria or bone pain, and had no history of cardiovascular and cerebrovascular diseases, diabetes, etc. The serum total prostate-specific antigen (tPSA) level was measured at 17.83ng/mL, and a digital rectal examination(DRE) revealed the presence of a hard nodule measuring approximately 1×1cm on the right lobe. The prostate magnetic resonance imaging (MRI) enhancement showed an abnormal signal in the migratory zone 9–11 points of the prostate, T2-weighted images indicated an equal/slightly hypointense lesion, and the PI-RADS score was 3 (**Figure 1A**). Chest computed tomography (CT) showed a solid nodule in the apical segment of the upper lobe of the right lung, with unclear boundaries and adhesion to the adjacent pleura, the size of which was about 2.8cm×2.3cm, and a suspicious metastatic lesion in the middle lobe (**Figures 1B, C**). A whole body bone scan (WBBS) and brain MRI were performed, both of which showed no evidence of tumor metastasis.

**Figure 2:**
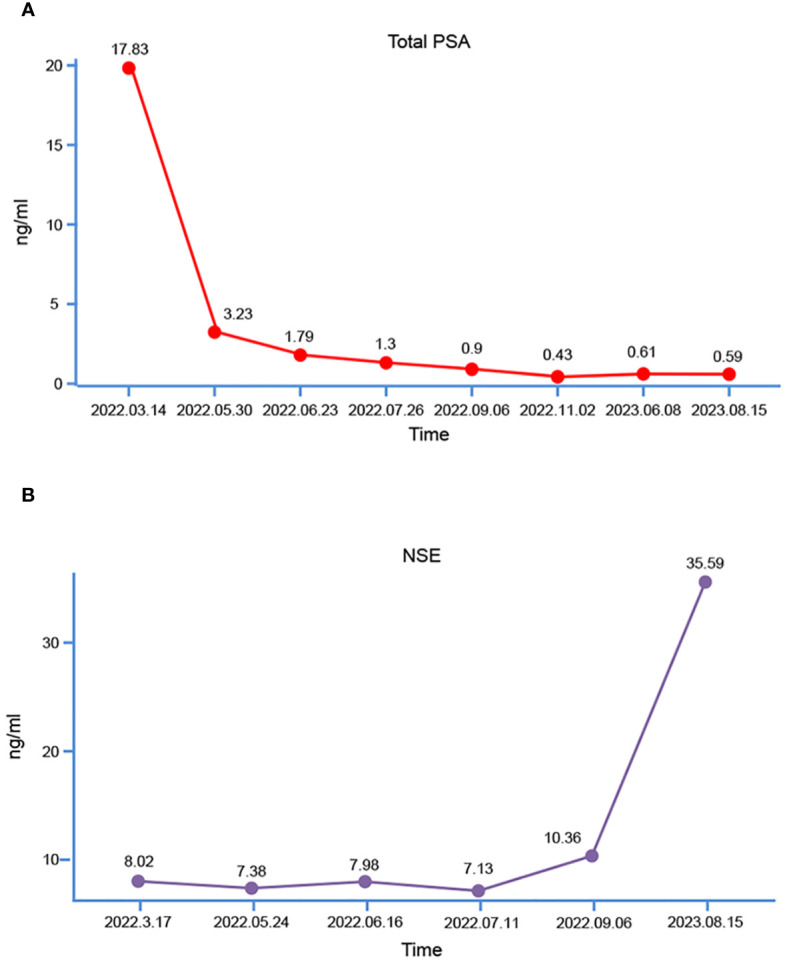
Trends in patient PSA and NSE. **(A)** Total PSA; **(B)** NSE.

Subsequently, the patient underwent transperineal magnetic resonance fusion color ultrasound prostate aspiration biopsy. Pathological examination showed that prostate adenocarcinoma, Gleason score was 7 (4 + 3), WHO/ISUP group 3 (**Figures 1D, E**). Then immediately began the androgen blockade treatment of goserelin acetate + bicalutamide (goserelin 3.6 mg per month and bicalutamide 50 mg daily). Simultaneously, the lung tumor was also considered malignant based on the chest CT report, and given that lung malignancies progress more rapidly than prostate cancer, the patient underwent thoracoscopic resection of the lung tumor on April 1th, 2022. Pathological examination revealed moderately differentiated invasive adenocarcinoma and metastatic prostate adenocarcinoma, the Gleason score was 8 (4 + 4). Following surgery, the patient underwent genetic sequencing, which indicated the absence of mutations in tumor suppressors such as TP53, BRCA2, PTEN, and CDKN2. Microsatellite instability (MSI) was detected as microsatellite stable (MSS), tumor mutation burden (TMB) was low, and poly (ADP-ribose) polymerase (PARP) inhibitor-related gene and PD-L1 expression tests were negative. Subsequently, the patient underwent 4 cycles of immunotherapy with sintilimab in combination with chemotherapy using paclitaxel and lobaplatin. During this period, myelosuppression occurred and recovered after timely treatment. During androgen blockade therapy, the patient underwent regular underwent PSA testing, which revealed a gradual decrease in PSA levels (**Figure 2A**). Furthermore, there was no observed increase in levels of neuron-specific enolase (NSE) (**Figure 2B**). In addition, a comprehensive evaluation including WBBS and chest CT was conducted, which did not reveal any indications of bone metastasis or recurrence of lung tumors. The patient reported only mild dysuria, without gross hematuria, bone pain, or weight loss.

**Figure 3:**
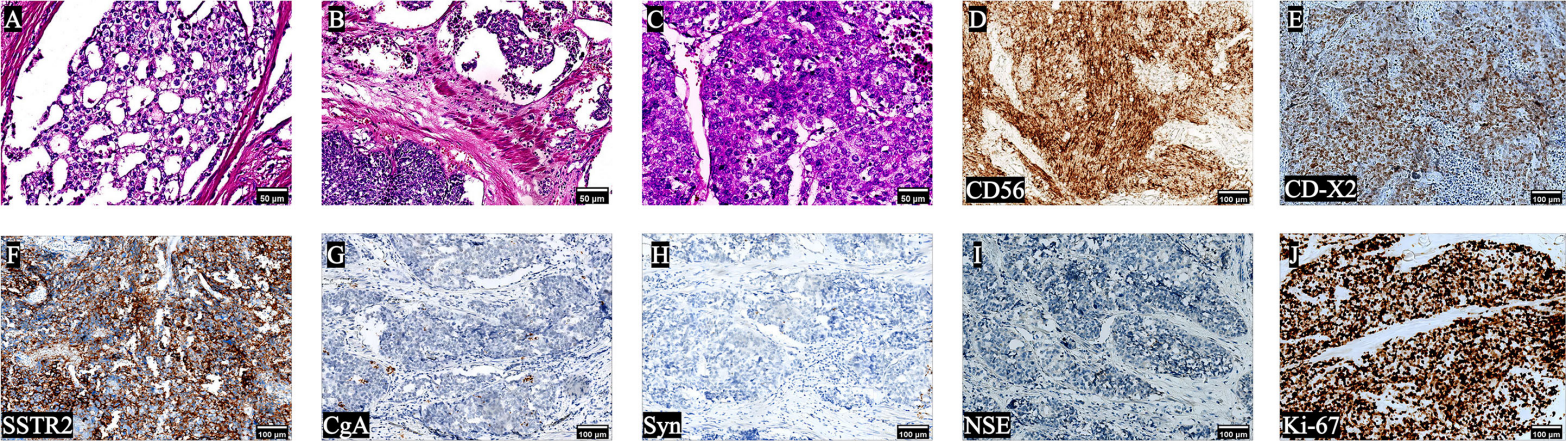
Pathological results of the patient after LRP. HE staining **(A-C)** showed that the simultaneous presence of adenocarcinoma and LCNEPC components; The cells exhibited large size, with palisade-like structures surrounding cell nests. The nuclei appeared large and deeply stained, indicating high mitotic activity, a low nuclear-to-plasma ratio, and extensive cell necrosis; IHC was positive for **(D)** CD56, **(E)** CDX -2, **(F)** SSTR2, and negative **(G-J)** for **(G)** CgA, **(H)** Syn, **(I)** NSE, **(J)** Ki-67 was strongly positive (90%) (10×).

One year after receiving ADT, after the patient fully communicates with us and expresses his willingness to operate, the patient returned to the hospital for LRP. Prior to LRP, the 18F-fluorodeoxyglucose positron emission tomography/computed tomography [(18F)FDG PET/CT] scan showed increased FDG metabolism in the right lobe of the prostate, compression of the bladder’s posterior wall, and no signs of metastasis in other organs were observed. The patient underwent LRP on June 13th, 2023. A cystoscopy was conducted prior to the operation, revealing an enlargement of the prostate with protrusion of the right lobe and middle lobe toward the bladder with invasion of part of the posterior wall of the bladder. No tumor was found in the bladder. During LRP, invasion of the right wall of the bladder by the right lobe and middle lobe of the prostate was observed, and the adhesion of the prostate to denonvillier fascia (DF) was unbounded. Consequently, a partial cystectomy was performed. Subsequent routine pathological examination indicated the presence of PRAD [Gleason score 8 (4 + 4)] with LCNEPC (approximately 10%) (**Figures 3A, B**), with LCNEPC identified in the bladder wall and the right bladder neck (**Figure 3C**). Hematoxylin-eosin (HE) staining revealed the presence of palisade-like structures surrounding the cell nests, characterized by large nuclei that exhibited deep staining, coarse chromatin, evident necrosis, and excessive mitotic activity (8). IHC analysis demonstrated positive expression of CD56, CDX-2, and SSTR2 (**Figures 3D-F**), while negative expression was observed for chromogranin A (CgA), synaptophysin (Syn), and NSE (**Figures 3G–I**), Ki-67 was strongly positive expression (+ 80%) (**Figure 3J**). After surgery, the patient did not receive further androgen blockade therapy and chemotherapy for personal reasons.

**Figure 4:**
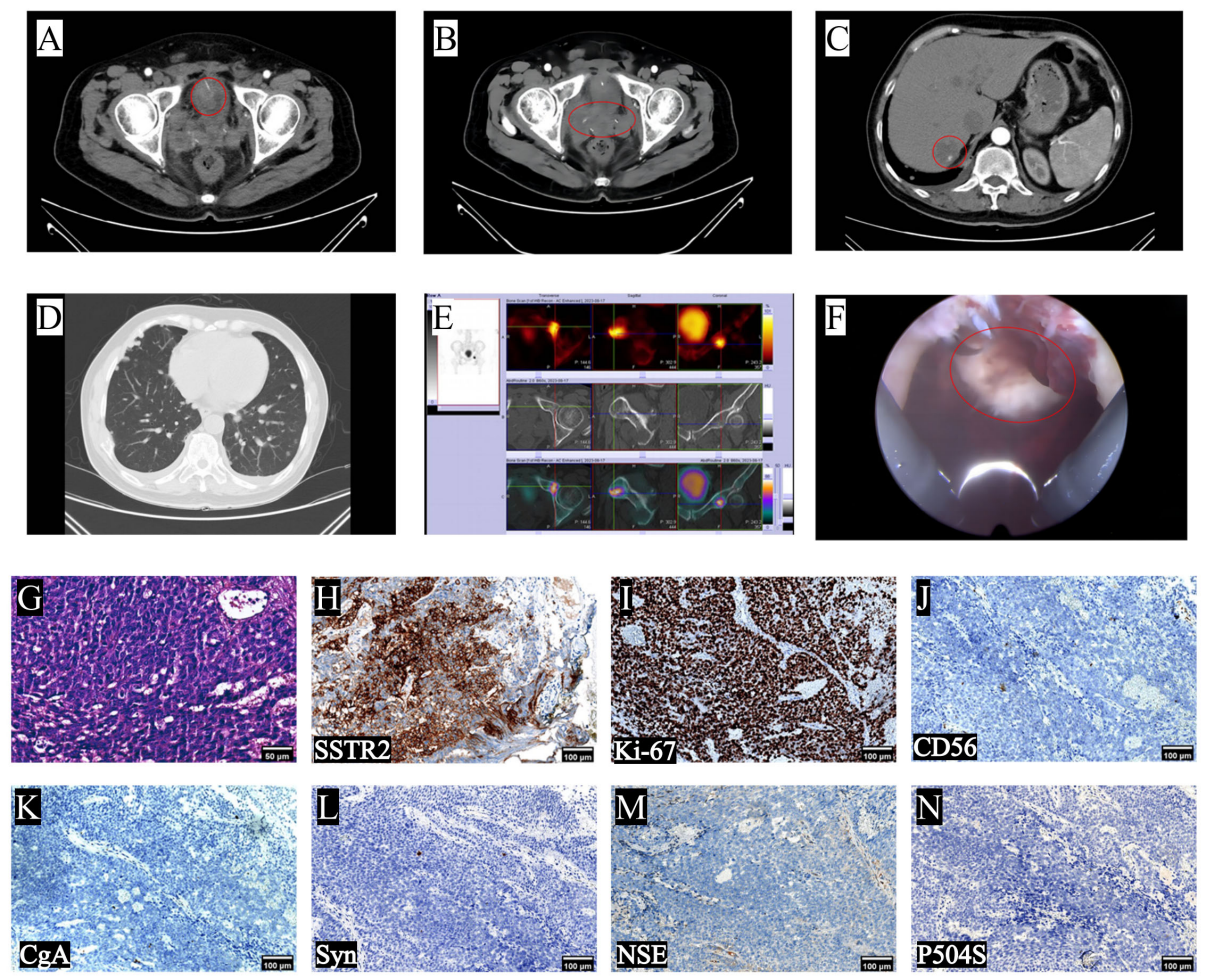
Imaging changes after patient progression, intraoperative conditions and postoperative findings. **(A, B)** CTU scans revealed the recurrence of bladder and pelvic tumors; **(C)** upper abdomen CT indicated hepatic metastasis; **(D)** chest CT showed multiple metastases; **(E)** WBBS indicated bone metastases; **(F)** intraoperative cystoscopy reveals tumor at 12 o’clock in the bladder; **(G)** typical LCNEPC presentation, no adenocarcinoma component, IHC was positive for **(H)** SSTR2 and **(I)** Ki-67 (90%), and negative for **(J)** CD56, **(K)** CgA, **(L)** Syn, **(M)** NSE and **(N)** P504S (10×).

Two months after undergoing LRP, the patient was readmitted to the hospital due to the presence of gross hematuria. The tPSA level was measured at 0.59ng/mL (**Figure 2A**), while the NSE level was recorded as 35.59ng/mL (**Figure 2B**). Computed Tomography urography (CTU) revealed the presence of enlarged masses in the region of the previous prostate and bilateral seminal vesicles, and significant space-occupying lesion on the right lateral wall of the bladder, considering tumor recurrence (**Figures 4A, B**). Intrahepatic nodules of small size were observed (**Figure 4C**). The chest CT scan revealed new multiple scattered nodules in both lungs compared to the previous scan (**Figure 4D**), indicating potential metastases. A WBBS indicated the presence of new metabolically active foci in the left acetabulum, as well as new osteolytic lesions in the pubic joint and bilateral ischium, suggesting bone metastases (**Figure 4E**). Additionally, the recurrence of intravesical tumors was considered as the likely cause of gross hematuria. On August 17th, 2023, a subsequent transurethral resection of bladder tumor (TURBT) revealed the presence of a tumor measuring 8 × 6 × 6cm (**Figure 4F**) on the top wall of the bladder, visible from the 12 o’clock position. Postoperative pathological examination indicated that the tumor exhibited characteristics consistent with LCNEPC without an acinar adenocarcinoma component (**Figure 4G**). IHC results demonstrated positive staining for SSTR2 and Ki-67 (90% +) (**Figures 4H, I**), negative staining for CD56, CgA, Syn, NSE, and P504S (**Figures 4J-N**).

Three weeks following the surgery, the patient underwent etoposide+ cisplatin chemotherapy (etoposide 150 mg daily for 3 consecutive days, and cisplatin 50mg for 3 consecutive days, and prepared to repeat every 4 weeks). The genetic sequencing report suggested that etoposide has a general effect with more pronounced side effects, while docetaxel and cisplatin exhibit greater efficacy with fewer side effects. Following thorough communication with the patient, the patient ultimately opted to proceed with the treatment plan of etoposide and cisplatin. After receiving chemotherapy, the patient developed severe myelosuppression, which was not relieved after treatment, multi-organ failure occurred, and the patient’s family refused further rescue measures. The patient was pronounced clinically dead on September 28^th^, 2023 (**Table 1**).

**Table 1:** Timeline of the patient process.

Time line in management	
March 14^th^, 2022	-First admission to hospital
March – July, 2022	-Completed prostate biopsy and thoracoscopic resection of the lung tumor - Completed 4 cycles of immunotherapy and chemotherapy
March 2022 – June,2023	-Androgen blockade therapy
June 13^th^, 2023	-Laparoscopic radical prostatectomy
August 17^th^, 2023	-Transurethral resection of bladder tumor
September, 2023	Received etoposide+ cisplatin chemotherapy
September 28^th^, 2023	-Pronounced clinically dead

## 3 Discussion

NE cells are typically found in prostate tissue and their numbers increase during puberty. These cells are less frequently observed in acini and do not express androgen receptors (AR) or secrete PSA (12). The differentiation of NE cells in the prostate can impede the apoptosis of prostate cancer cells. Consequently, the extent of NE differentiation in the majority of prostate cancers is associated with heightened tumor invasiveness, rapid disease progression, and poor prognosis (13).

Adenocarcinoma is the most prevalent pathological subtype of prostate malignant tumors, and the standard treatment approach involves ADT and antiandrogenic therapy. The majority of neuroendocrine cancers, including LCNEPC, arise in the context of long-termed ADT treatment (5). Because ADT does not completely eliminate NE cells (14). According to the review of the literature, 12 cases of prostate adenocarcinoma differentiated into LCNEPC after being treated with ADT. The mean serum PSA at the time of initial diagnosis of prostate adenocarcinoma in 10 patients was 28.05 ng/mL (range 0–90ng/mL). The mean duration of ADT treatment was 3.5 years (range 2–9 years). The average time from the first diagnosis of prostate cancer to the diagnosis of LCNEPC was 4.7 years (range 2–9 years). Among the patients diagnosed with LCNEPC, chemotherapy was administered to 7 individuals, and the final outcome in 11 patients was death or loss to follow-up (15). Currently, only one patient, who harbored a somatic BRCA2 mutation, survived after receiving treatment with the Rad3-related protein (ATR) Inhibitor (M6620) in conjunction with gemcitabine, cisplatin, and etoposide, achieving a progression-free survival (PFS) of 20 months (16).

Based on the available literature, a total of 12 cases of primary LCNEC of the prostate were reported with an average overall survival of 21.5 months(range 7–54 months) (15). Patients with a diagnosis of pure LCNEPC who received a chemotherapy regimen of etoposide + cisplatin after diagnosis still had an extremely poor prognosis, with a mean survival of 7.3 months (5, 17). Among the patients diagnosed with NE carcinoma-acinar adenocarcinoma mixed type who received chemotherapy in combination with ADT and the median survival time was significantly longer than that of pure LCNEPC patients (18, 19). Another patient diagnosed with primary LCNEC combined with adenocarcinoma received ADT combined with chemotherapy with etoposide + cisplatin, and after terminating the chemotherapy was treated with abiraterone because of the high metastatic risk, and the patient was followed up for 20 months without progression (20). However, untreated cases of LCNEPC tend to progress rapidly, ultimately resulting in mortality (21). In an unpublished case, the tumor invaded the bladder neck and the patient was initially treated with cisplatin and etoposide, followed by the addition of pembrolizumab and Leuprolide, which did not impede the tumor’s progression. Subsequently, the patient underwent treatment with docetaxel (75 mg/m^2^) and prednisone. After 5 cycles, imaging revealed improvement of the sclerotic lesions, and no new metastatic lesions were detected, with the PSA value measuring less than 0.01ng/mL. It shows that docetaxel and prednisone may have efficacy in the treatment of LCNEPC (15). In our case, the patient was not treated with docetaxel, making it impossible to know whether the patient would have had a better outcome and longer survival.

Two possible mechanisms have been proposed to explain the occurrence of LCNEPC based on the appealing two scenarios. Firstly, it is suggested that patients with adenocarcinoma may develop LCNEPC after long-term ADT treatment, This could be attributed to the selection pressure exerted by ADT, which affects the cloning progression of non-NE cells. Consequently, the loss of androgen receptor (AR) expression occurs, leading to the emergence of NE cells that do not express AR. This process is known as transdifferentiation (5, 22). Additionally, *in vitro* studies of the prostate cancer cell line LNCaP have demonstrated a decrease in AR expression in cultured cells lacking androgens (22). RaPa et al (23) believed that the NE differentiation of prostate cancer is a dynamic process that is influenced by androgen deprivation. This process involves the participation of various cytokines and growth factors in the acquisition of neuroendocrine phenotypes. While the evidence for the transdifferentiation of LCNEPC remains uncertain, the presence of NE carcinoma-acinar adenocarcinoma mixed type serves as a strong indication of transdifferentiation (8). In this case, both adenocarcinoma and LCNEPC were detected simultaneously during LRP one year after ADT treatment, However, when a recurrent bladder tumor was resected two months later, only LCNEPC was observed. This mechanism is consistent with what has been observed in several clinical cases, including our case. The second mechanism suggests that LCNEPC can develop in the absence of prior ADT treatment through the direct malignant transformation of normal prostate NE cells, as reported in a limited number of cases (6, 24). However, the precise mechanism underlying this process necessitates further investigation.

In this case, the patient was diagnosed with LCNEPC one year after receiving ADT, a notably shorter than any previously reported in scholarly literature. Had the patient not undergone LRP, the diagnosis of LCNEPC would have been unattainable, potentially resulting in a more unfavorable prognosis. Timely diagnosis of LCNEPC is of paramount importance, but which patients will develop into LCNEPC is unpredictable. The definitive diagnosis of LCNEPC hinges on the pathological examination. Obtaining serial biopsy samples at multiple time points between initiation of ADT and the diagnosis of NEPC can provide an in-depth insight into disease progression, and is one of the methods for early diagnosis, and guide the timely revision of the treatment strategies (25). However, executing this procedure in a clinical setting remains challenging. Through our case, we believe that detecting the changes of NSE level in the blood may also be a valuable diagnostic tool for NEPC. As illustrated in **Figure 2B**, there was an observable increase in patients’ NSE level in September 2022 (5 months after initiating ADT therapy). Despite remaining within the normal range, it was not taken seriously until the patient was tested again 2 months after LRP, and it was suggested that the NSE level had been significantly increased. Consequently, it is recommended that patients undergo regular monitoring of NSE level, and prompt diagnosis should be pursued upon detection of an increasing trend. In the diagnosis of LCNEPC, the following features need to be met in terms of morphology: 1) The cells are large and polygonal, with large and deeply stained nuclei, abundant cytoplasm, and low nuclear/cytoplasmic ratio; 2) Typical cell nests surrounded by a palisade-like structure; 3) A high mitotic rate and extensive cell necrosis are evident. In IHC, NE markers such as Syn, CgA, and CD56 are utilized, with at least one of these markers needing to be positive (8). However, in a reported case of SCNC of the prostate, the patient exhibited negative results for CD56, NSE, Syn, and CgA, and was finally diagnosed as SCNC according to its clinical manifestations, auxiliary examinations, and cellular morphological features (26). In our case, in the pathologic diagnosis after TURBT, the IHC results indicated negative findings for CD56, NSE, Syn, and CgA, and only positive for SSTR2. Therefore, we posit that in cases where morphology aligns with neuroendocrine carcinoma and common markers such as CgA, Syn, CD56, and NSE are negative, it is necessary to further enhance the identification of other markers, such as SSTR2 (27), CDX-2 (28), enhancer of zeste homologue 2 (EZH2) (29), heterochromatin protein 1α (HP1α) (30) and forkhead box A1 (FOXA1) (31) to further achieve a more precise diagnosis of neuroendocrine carcinoma and establish a foundation for subsequent treatment. These molecular markers that may be used in the diagnosis of NEPC.

Additionally, the expression of PSA serves as a distinguishing criterion between adenocarcinoma and neuroendocrine cancer, as NEPC rarely exhibits PSA expression (32). It is important to note that certain cases of NE carcinoma-acinar adenocarcinoma mixed type may express PSA, while high-grade acinar adenocarcinoma can also express neuroendocrine markers (33). Hence, the morphological and IHC diagnosis and differentiation are crucial for the diagnosis of NE carcinoma. In this case, the patient experienced systemic multi-organ metastases two months after undergoing LRP. Notably, the level of PSA did not increase but rather decreased compared to the pre-LRP level, and the patient did not receive ADT during this period. It is also confirmed that LCNEPC does not express AR or PSA.

The treatment approach for LCNEPC significantly differs from that of typical adenocarcinoma due to the lack of androgen receptor expression and resistance to hormone therapy exhibited by most NEPCs (34), From the studies reported to date, may only derive benefits from ADT in LCNEPC patients contains adenocarcinoma component (18, 19, 35). Currently, there is a lack of standardized treatment plans or guidelines for these patients. While active surgery can be performed for patients with resectable lesions, the majority of patients are diagnosed at an advanced stage of the disease, resulting in missed opportunities for surgical intervention. Consequently, chemotherapy becomes the primary treatment option. Typically, platinum-based chemotherapy regimens, with etoposide + cisplatin being the most commonly selected regimen, are employed (5, 9). In cases where etoposide proves ineffective, regimen docetaxel, gemcitabine + platinum may be chosen as an alternative (11, 36). In this case, the patient was diagnosed with LCNEPC following LPR, and chemotherapy was not initiated initially but was only contemplated after the occurrence of hematuria and reoperation 2 months later. Therefore, we recommend that chemotherapy or other treatments be commenced at the time of initial diagnosis in future patients presenting with similar conditions. If there is a combination of adenocarcinoma component with metastatic lesions, the consideration of ADT treatment in conjunction with novel hormonal therapies like apalutamide, rezvilutamide, darolutamide, etc., alongside the initiation of chemotherapy, is recommended. Furthermore, the initial genetic sequencing report did not identify any gene mutations, and no further genetic sequencing was performed at the time of the subsequent diagnosis of LCNEPC, so it was not possible to know whether mutations in genes such as TP53 and BRCA2 had occurred, and it was not possible to formulate a treatment strategy based on the results of the genetic sequencing. It is advised for LCNEPC patients to undergo genetic sequencing, although it is important to acknowledge that the genetic sequencing report merely serves as a treatment reference and does not dictate the selection of therapy for patients. Furthermore, it is possible for different genetic sequencing companies to yield varying results. Therefore, more studies on this disease is still needed to provide a theoretical basis for the selection of treatment options.

Meanwhile, it was mentioned earlier that a patient with the BRCA2 somatic mutation who received a combination of M6620 and etoposide + cisplatin + gemcitabine chemotherapy had a significantly better prognosis than other cases (16). M6620 is an emerging and potent ataxia telangiectasia mutated and Rad3-related protein (ATR) inhibitor (37). This case suggests that patients with neuroendocrine prostate cancer may be considered in combination with ATR inhibitors on the basis of chemotherapy, especially in patients with mutation genes involved in homologous recombination repair pathway.

The metastatic site of LCNEPC closely resembles that of PRAD, primarily involving bone, liver, lungs, and lymph nodes (5). However, instances of brain metastasis have also been reported (38), albeit as a rare occurrence. In the present case, the patient exhibited metastases in the lungs, liver, lymph nodes, and bone, as well as a recurrence of pelvic and bladder tumors, all within a mere two-months. We intended to conduct a PET/CT or brain MRI brain MRI scan to assess the presence of brain metastasis; however, the patient declined the aforementioned examination due to financial constraints. Consequently, the patient’s condition of brain remains unknown, although he did not exhibit symptoms such as headache, nausea, or blurred vision. It is worth noting that the sample size of patients is limited, and further investigation is necessary to elucidate the specific mechanisms underlying LCNEC metastasis.

LCNEC is characterized by high invasiveness and a bleak prognosis. Evans et al. (5) reported that 6 patients survived for an average of 7 months after diagnosis of LCNEPC, and Marcus et al. (39) reported a median survival duration of 10 months in patients diagnosed with NE tumors, accompanied by a 5-year overall survival rate of 12.6%. Tanaka et al. (40) reported that patients with NEPC have the following typical clinical course: 1) short survival time after recurrence; 2) PSA level does not increase after recurrence; 3) metastatic sites resembling those of adenocarcinoma. The clinical trait presented in this case is consistent with the above viewpoints. Ultimately, the patient succumbed to systemic multi-organ failure caused by post-chemotherapy myelosuppression, leading to his demise three and a half months after being diagnosed with LCNEPC.

To sum up, LCNEPC represents a rare form of prostate cancer characterized by a high level of malignancy, a propensity for early metastasis, and a poor prognosis. This study presents a case initially diagnosed as prostate adenocarcinoma, which subsequently transformed into mixed NE carcinoma-acinar adenocarcinomas following treatment with ADT and metastasized to multiple organs within two months of LCNEPC diagnosis. The patient succumbed to the disease 3.5 months later. The diagnosis of LCNEPC relies on HE+ IHC staining, and platinum-based chemotherapy serves as the primary treatment modality. Further investigation into the molecular biological characteristics of this disease is warranted. Although there are epigenetic regulators that are altered in NEPC, whether this affects genetic predisposition, including the molecular biology characterizing the disease, requires further study.

## Data availability statement

The raw data supporting the conclusions of this article will be made available by the authors, without undue reservation.

## Ethics statement

The studies involving humans were approved by Chongqing General Hospital, Chongqing University. The studies were conducted in accordance with the local legislation and institutional requirements. The a member of the patient's family provided their written informed consent to participate in this study. Written informed consent was obtained from the individual(s) for the publication of any potentially identifiable images or data included in this article.

## Author contributions

MX: Writing – review & editing, Writing – original draft, Resources, Conceptualization. WT: Data curation, Writing – review & editing. XX: Writing – review & editing. XP: Writing – review & editing. FY: Writing – review & editing, Funding acquisition, Conceptualization.
